# Social and environmental risk factors for dengue in Delhi city: A retrospective study

**DOI:** 10.1371/journal.pntd.0009024

**Published:** 2021-02-11

**Authors:** Olivier Telle, Birgit Nikolay, Vikram Kumar, Samuel Benkimoun, Rupali Pal, BN Nagpal, Richard E. Paul

**Affiliations:** 1 Géographie-cités, Université Paris-1 Panthéon-Sorbonne, Paris, France; 2 Centre for Policy Research, Dharam Marg, Delhi, India; 3 Mathematical Modelling of Infectious Diseases, Institut Pasteur, Centre National de la Recherche Scientifique, Paris, France; 4 National Institute of Malaria Research, Sector 8, Dwarka, Delhi, India; 5 Centre de Sciences Humaines, UMIFRE 20 CNRS-MAE,Delhi, India; 6 Institut Pasteur, Functional Genetics of Infectious Diseases Unit, Paris, France; University of Heidelberg, GERMANY

## Abstract

Global urbanization is leading to an inexorable spread of several major diseases that need to be stemmed. Dengue is one of these major diseases spreading in cities today, with its principal mosquito vector superbly adapted to the urban environment. Current mosquito control strategies are proving inadequate, especially in the face of such urbanisation and novel, evidence-based targeted approaches are needed. Through combined epidemiological and entomological approaches, we aimed to identify a novel sanitation strategy to alleviate the burden of dengue through how the dengue virus spreads through the community. We combined surveillance case mapping, prospective serological studies, year-round mosquito surveys, socio-economic and Knowledge Attitudes and Practices surveys across Delhi. We identified lack of access to tap water (≤98%) as an important risk factor for dengue virus IgG sero-positivity (adjusted Odds Ratio 4.69, 95% C.I. 2.06–10.67) and not poverty *per se*. Wealthier districts had a higher dengue burden despite lower mosquito densities than the Intermediary income communities (adjusted Odds Ratio 2.92, 95% C.I. 1.26–6.72). This probably reflects dengue being introduced by people travelling from poorer areas to work in wealthier houses. These poorer, high density areas, where temperatures are also warmer, also had dengue cases during the winter. Control strategies based on improved access to a reliable supply of tap water plus focal intervention in intra-urban heat islands prior to the dengue season could not only lead to a reduction in mosquito abundance but also eliminate the reservoir of dengue virus clearly circulating at low levels in winter in socio-economically disadvantaged areas.

## Author summary

Identifying disease hotspots and individual risk factors for dengue can enable targeted intervention strategies. We conducted combined serological, entomological and socio-economic surveys across 18 areas within Delhi, taken from the total 1280 colonies (i.e. the administrative units of reference in Delhi) for which we classified their socio-economic typology. We additionally performed a Knowledge, Attitudes, Practices survey at a household level within the most socially disadvantaged sub-districts. Finally, we mapped all the winter dengue cases to 250 m x 250 m units along with their winter mean temperatures. We found that access to tap water was an important risk factor for exposure to dengue virus (DENV) and this was confirmed even within the socially disadvantaged sub-districts. The Wealthy colonies had a high burden of DENV infection despite low mosquito densities, likely linked to their connectedness through daily human mobility. The winter burden of dengue occurred majoritarily in the socio-economically disadvantaged colonies, which also have higher mean temperatures and urban heat islands. Improved access to tap water could lead to a reduction in dengue, not only for those directly affected but for the general population. Targeted intervention through mosquito control in winter in the socially disadvantaged areas could offer a rational strategy for optimising control efforts.

## Introduction

We are currently bearing witness to an inexorable rise in mosquito-borne viruses in particular those transmitted by the urban mosquito vector, *Aedes aegypti*. Currently, it is estimated that 3.5 billion people are at risk of the most widespread arbovirus, dengue virus (DENV), and with the increasing rate of urbanisation, most notably in the tropics, this figure will be rapidly surpassed [1]. It is widely agreed that mosquito control will play the major role in mitigating against dengue and other arboviral diseases and that currently the approaches used are outdated and ineffective [2]. Over the past 100 years, the most successful dengue control strategy has been based on the focal insecticide treatment in and around mosquito oviposition sites. That approach eradicated *Aedes aegypti* from 22 countries in the Americas during the 1950s and 1960s [3], but required enormous human resources. Today, the scale of recent urban expansion negates such an approach. Other than the massive vector control response to the Cuban 1981 epidemic [4], the only other example of temporally successful control of dengue was by Singapore that combined source reduction with education and law enforcement [5]. Subsequent changes from vector control to dengue case detection and fumigation in Singapore may have been less effective and contributed to the subsequent re-occurrence of dengue epidemics [5]. Novel control strategies are clearly required, not least to optimise the use of the meagre resources dedicated to the Public Health sector within any city.

The universally recognised link between sanitation and disease offers a key but as of yet unexploited point of leverage. A wealth of studies has examined the association of socio-economic status and risk of dengue, but with conflicting outcomes as to whether dengue is really a disease of poverty [6–9]. Social inequality is often associated with high human density, inadequate infra-structure and poor environmental hygiene. Whereas the former enables rapid local spread of DENV generating dengue clusters [10,11], the latter provide an ideal environment for the mosquito to breed. Absence or intermittence of piped water and the subsequent need for water storage generate ideal oviposition sites and solid waste provides the same during the seasonal rains [12–14]. These factors are often found to be associated with risk of dengue, but not systematically so [6,15]. Identifying factors associated with risk of dengue is, however, challenging, not least because of the high but variable proportion of inapparent infections, increasing evidence for short-term heterotypic cross-protective immunity and the potential occurrence of herd immunity [16,17]. However, an additional complication is the role of human mobility in ferrying the virus from places of high environmental risk throughout the city [18–20]. The identification of the source of infection and the subsequent socio-spatial structure of the intra-urban spread of DENV would clearly aid the local public health services to better allocate resources. The Indian metropolis of Delhi houses 16.7 million inhabitants with high social inequality distributed heterogeneously throughout the city [21]. Delhi has recorded dengue cases every year since 1996 with seasonal transmission occurring from July until November, after which time ambient temperatures drop, curtailing transmission. Increasing population density with concomitant vertical, dense building development, combined with the lack of vegetation especially within the socially disadvantaged areas is, however, contributing to urban heat islands that are most pronounced during the winter, thereby extending the transmission period [14]. Combining surveillance case mapping, prospective serological studies, as well as year-round mosquito abundance detection within a socio-economic context, this study aims to assess the association of socio-economic typology with risk of dengue and identify specific risk factors.

## Methods

### Ethics statement

Written consent to participate in the study was obtained from all participants and ethical approval was granted by the ethics committees of the Indian Council for Medical Research, India (N° TDR/587/2012-ECD-11, 10 December 2012) and Institut Pasteur, France (N° 2011–20, 29 April 2011). If human subjects were not adult, a parent or guardian of the child provided written informed consent on their behalf. Patient data were anonymized.

### Study sites and study population

Delhi is comprised of 1280 colonies covering a wide disparity of socio-economic status (SES) distributed throughout the city. Colonies are administrative spatial units that are used for property tax collection and public investment. Based on several variables assessing the level of infrastructure and access to urban services, property tax varies from one colony to another through a Unit Area Value system [22]. Integrating data on land use, population density and property tax ranking for each colony in a GIS enables an estimation of local socio-environmental heterogeneity. SES of each colony was determined prior to the study in 2011 by the municipality of Delhi in collaboration with the Survey of India, after having conducted a survey to determine local characteristics. Each colony was allotted a property tax score (out of 100) composed of 10 criteria (criteria noted out of 10): 1) access to physical urban infrastructures (such as water, quality of road) and 2) social infrastructures (such as presence of a hospital), 3) the age of the colony, 4) its type (approved, non-approved, recently approved, etc.), 5) economic status of residents, 6) cadastral (price of the land) and 7) rent values of the property, 8) access to commercial centers, 9) colony location in Delhi and 10) Road accessibility. Each criterion of the property tax system is decomposed into three levels: 10 if the rank is high, six for middle and three for very low. This generated a wide range of property tax scores (S1 Fig). In addition to the socioeconomic variables, we added detailed built-up data from the Global Human Settlement database [23] in order to estimate the population at 250 m x 250 meters. This dataset provided by the European Union allows access to very high-resolution data (up to 38 m) for all the countries in the world. Combined with the final score of all 1,280 colonies in Delhi, this technique enabled us to create our SES typologies at a very fine social and spatial resolution using the Jenks optimization method [24]. The following SES typologies were obtained: Deprived High Density (HD) (colonies below 40 points on the property tax scale and more than 30,000 individuals/km^2^), Deprived (below 40 points and less than 30,000 individuals/km^2^), Intermediary (between 40 to 65 points), Wealthy (above 65 points), Urban Village (separate status of property tax), Peripheral (below 500 individuals/km^2^)(S1 and S2 Tables). New Delhi Municipal Corporation (NDMC) is an additional classification, having no property tax status. This absence of property tax score could be an issue to compare this category with others and was not included in the sero-survey. NDMC is not highly populated, being the area dedicated to administration (only 2% of the population of Delhi reside in the area, see S2 Fig). In addition to SE typology classification, we retrieved information on population size, population density, access to tap water (%), percent of houses with greater than five people, condition of house (good, intermediate, dilapidated), waste water outlet (connected to closed drain, open drain, no drain), latrine (Y/N), source and place of drinking water for every study colony. Categories were generated from these percentages or N by using Jenks optimization method [24] (S3 Table), which enables a reduction of the variance within a category and maximization of the variance between categories. For all socio-economic information we used official data provided by the Census of India 2011 [21].

### Colony selection and sample size calculations

**Fig 1:**
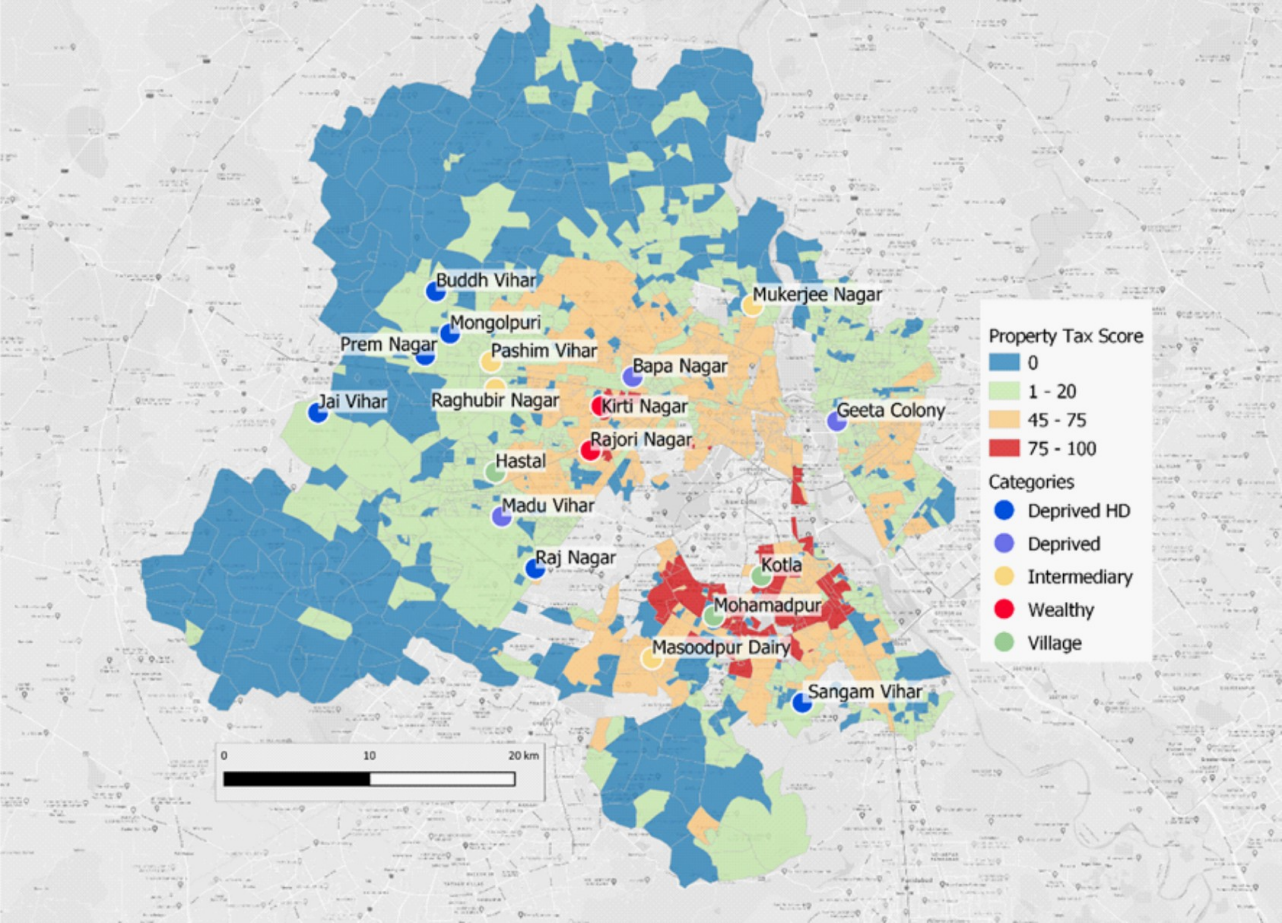
Map of 18 selected study sites within Delhi.

Within logistical and financial limitations, the aim was to request permission from local government authorities to select and recruit from at least three colonies of each SE typology, providing a minimal measure of intra-typology variation in sero-prevalence rates. The colonies from each SES were selected randomly, but selected within 1h30 drive from the National Institute of Malaria Research for logistical reasons. This resulted in a total of 18 colonies: six Deprived HD, three Deprived, four Intermediary (aka Medium), two Wealthy and three Village colonies (Fig 1).

Within each colony, we aimed to recruit at least one hundred participants from randomly selected houses within each colony and within 200 m from an index dengue case identified through the surveillance system. This number was based on the assumption that the highest sero-prevalence rate would be 40% positive and thus 100 participants would enable us to detect a 50% difference in sero-prevalence in other colonies. Here we assumed that all colonies were behaving independently, irrespective of whether they had the same SE typology. Assuming no intra-typology variation in sero-prevalence rates, 300 individuals would enable us to detect differences as low as a 33% decrease. The sample size was calculated using the following standard formula when considering proportions (incidence rate),

$$n=2{\left(Z\alpha +Z1-\beta \right)}^{2}p\left(1‐p\right)/{\left(p0‐p1\right)}^{2}$$

Where n is the required sample size per site, Zα and Z1-β are constants set by convention according to the accepted α error and if a one-sided or two-sided effect. p0 is proportion infected in site A and p1 proportion infected in site B and p = (p0+p1)/2. Assuming a *p* < 0.05 as acceptable and a study with 90% power; the following constant values are: Zα is 1.96 (two-tailed); Z1-β is 1.2816.

### Epidemiological and entomological data

#### Surveillance case data

As detailed in Telle et al. 2016 [10], dengue cases reported through the Delhi surveillance system (sentinel network consists of 38 public and three private hospitals) in 2008, 2009 and 2010 were geo-localised at a scale of 250 m x 250 m. The dengue cases were additionally geo-localised for the winter period from 2013 to 2015, when dengue cases were announced in winter for the first time. Dengue cases had been confirmed for the presence of IgM antibodies against DENV by MAC ELISA using a kit prepared by the National Institute of Virology, Pune, India as an integral part of the National Vector Borne Disease Control Programme. These confirmed cases were geocoded with QGIS [25].

#### Serological and entomological surveys

A sero-epidemiological study was performed from September to November (2013) on 2,107 individuals from the 18 pre-selected colonies using SD Duo Bioline rapid diagnostic tests (RDT) to detect IgG, IgM and NS1. Individuals were recruited in the immediate vicinity (200 m radius) of confirmed dengue index cases identified through the surveillance system. Entomological surveys were carried out in all colonies every month to inspect all water-filled containers present in and around houses. Each house was visited on only one occasion (month). The larval collections were made from each locality with the help of flash-light, by dipping and pipetting methods. Mosquito larval indices (house index and container index) were measured in all the colonies throughout the year (June 2013-May 2014) on a monthly basis. House index is the percentage of houses found to be positive for larvae and container index, the percentage of containers positive per house.

#### Family-based study of socio-economic factors and dengue sero-positivity

In addition, we performed a questionnaire based Knowledge, Attitudes and Practices (KAP) study in individuals from 5 colonies: Deprived HD (3 chosen randomly among the 6 colonies studied for sero-prevalence) and Deprived categories (2 chosen randomly among the 3 colonies studied for sero-prevalence) (S4 Table). Dengue sero-positivity was measured as above. A total of 338 individuals from 152 families were surveyed. We aimed to recruit a minimum of 30 houses in each colony. The acceptance rate from the 50 houses approached in each colony was very variable. In the end we achieved recruitment of a total of 227 individuals in three Deprived HD colonies (within 29 + 40 + 17 households) and 111 individuals in two Deprived colonies (within 37 + 29 households). All houses were different from the first survey above.

#### Temperature mapping

At-satellite brightness temperature was retrieved from LANDSAT 8 TIRS (band 10), applying the method described by Walawender et al. 2014 [26](the thermal image was taken on November 15, 2013 at around 5:00 AM). Land surface temperature was computed using the correction equations given by the same authors (land surface emissivity and atmospheric bias corrections) (See [14], for further details). Surface temperatures were obtained at a 38 m scale and then aggregated to the 250 m grid level.

### Statistical analysis

Logistic regression was used to analyze the association of risk factors with mosquito (house and container indices) and serological outcomes. For association with mosquito indices, a Generalized linear model (GLM) was fitted with the following risk factors considered: SE typology, month of year and tap water access category. For serological analyses, considered risk factors were age class, gender, colony SE typology, colony ID and the colony level socio-economic variables; a Generalized Linear Mixed Model (GLMM) logistic regression was fitted with house of the individual as the random variable to account for repeated measures (multiple individuals) from the same house. Initially all variables were tested in a univariate analysis and then all variables having a P value inferior to 0.25 were tested in a multivariate analysis, generating Odds Ratios adjusted for other fitted parameters. Model simplification in the multivariate analysis was carried out fitting all variables and removing non-significant variables sequentially.

For the subset of colonies where KAP surveys were carried out, analyses of IgG sero-positivity using information from the KAP questionnaire were performed by fitting a GLMM logistic regression with family of the individual as the random variable to account for repeated measures (multiple individuals) from the same family. Initially all variables were tested in a univariate analysis and then all variables having a P value inferior to 0.25 were tested in a multivariate analysis (S5 Table), generating Odds Ratios adjusted for other fitted parameters. Model simplification was carried out fitting all variables and removing non-significant variables sequentially.

For the analysis estimating the association of SE typology with risk of winter dengue cases, analysis was performed at a finer scale than colony, that of 250 m x 250 m units. A GLM loglinear regression was fitted with Log Population size of each 250 m x 250 m unit as an offset and SE typology fitted as an explanatory variable. In light of the increasing temperatures with population density, we sub-divided Deprived into Low (Deprived LD: <5,000 individuals/km^2^) and Medium (Deprived MD: 5,000–30,000 individuals/km^2^) densities and Intermediary into Intermediary High Density (>20,000 individuals/km^2^) and Intermediary (<20,000 individuals/km^2^) categories. In all cases, a dispersion parameter was estimated to take into account any over-dispersion in the data. Wald statistics, which approximate to a χ^2^ distribution, were established. All analyses were performed in Genstat version 15 [27].

## Results

Of 2107 individuals tested across the 18 colonies and within the vicinity of a dengue index case, 160 (7.6%) were found to be positive for either IgM or NS1, evidence for recent or current infection respectively. All variables were significantly associated with IgM/NS1 positivity in the univariate analyses at P<0.25. In the multivariate analysis, Wealthy colonies had higher risk of recent infection than Deprived HD (Table 1). Wealthy colonies also had higher risk of recent infection than Intermediary colonies (aOR 8.05 95% C.I. 2.16–30.06, P = 0.0033). Otherwise there were no differences among the other SE typologies (Table 1). Colonies with very poor access to tap water (<61% of houses had access) were associated with higher sero-positivity (aOR 3.80, 95% C.I. 1.25–11.58, P = 0.025). Such very poor access to tap water only occurred in Deprived HD colonies; one Deprived HD colony, however, had very good access to tap water. Young age (0–14 yrs) increased risk of positivity as compared to adults (>24 years old) (Table 1). Large population size (>3000 individuals) was associated with low IgM/NS1 sero-positivity, but population density was not (P = 0.34). No other environmental risk factors were found to be associated (House condition P = 0.40; Waste water outlet P = 0.42; Latrine P = 0.92; House >5, P = 0.91).

**Table 1:** Association of parameters with recent and/or primary dengue infection (IgM and/or NS1+ using SD Bioline Duo RDT) in the multivariate analysis.

Parameter	Tested	Positive	aOR	95%CI	P value
**Age group**					
0-14y	692	67	**2.03**	1.55–2.66	<0.001
15-24y	352	25	1.08	0.74–1.56	0.370
25+y	1054	68	REF		
**SES typology**					
Wealthy	162	6	**6.27**	1.36–29.98	0.025
Intermediary	410	19	0.78	0.22–2.73	0.370
Village	348	26	1.30	0.29–5.84	0.376
Deprived	396	27	3.75	0.78–18.13	0.103
Deprived HD	791	82	REF		
**Colony level Tap Water Access %**					
24–60	700	77	**3.80**	1.25–11.58	0.025
61–92	430	25	0.35	0.10–1.19	0.096
93–98	557	38	1.48	0.54–4.06	0.298
>98	420	20	REF		
**Population Size (N)**					
1000-<2000	731	68	**9.44**	4.48–19.88	<0.001
2000-<3000	642	69	**9.17**	4.50–18.68	<0.001
≥3000	734	23	REF		

aOR–adjusted Odds Ratio; 95%CI- 95% Confidence Intervals.

Previous exposure to any of the four dengue serotypes (sero-positive IgG) revealed comparable results, with younger age (0–14 years), Intermediary and Deprived typologies being associated with lower risk and ≤98% access to tap water being associated with higher risk (Table 2). Combining all three tap water categories of ≤98% access and comparison against the >98% category revealed that those with ≤98% tap water access had 4.69 increased odds of dengue (OR 4.69 95% CI 2.06–10.67). Gender was not found to be associated with IgG positivity (P = 0.76). Population density was again not found to be associated with increased risk of sero-positivity (P = 0.23) and no other environmental variables were significantly associated.

**Table 2:** Association of parameters with previous dengue infection (IgG) in the multivariate analysis.

Parameters	Tested	Positive	aOR	95%CI	P value
**Age group**					
0-14y	692	211	**0.60**	0.45–0.80	<0.001
15-24y	352	125	0.73	0.52–1.03	0.108
25+y	1054	375	REF		
**SES typology**					
Wealthy	162	50	0.93	0.44–1.97	0.844
Intermediary	410	67	**0.32**	0.14–0.73	0.007
Village	348	101	0.45	0.15–1.35	0.157
Deprived	396	156	**0.32**	0.10–0.98	0.047
Deprived HD	791	337	REF		
**Colony level Tap Water Access %**					
24–60	700	311	**4.69**	2.06–10.7	<0.001
61–92	430	161	**2.45**	1.24–4.67	0.040
93–98	557	151	**2.29**	1.14–4.59	0.043
>98	420	88	REF		

aOR–adjusted Odds Ratio; 95%CI- 95% Confidence Intervals.

**Fig 2:**
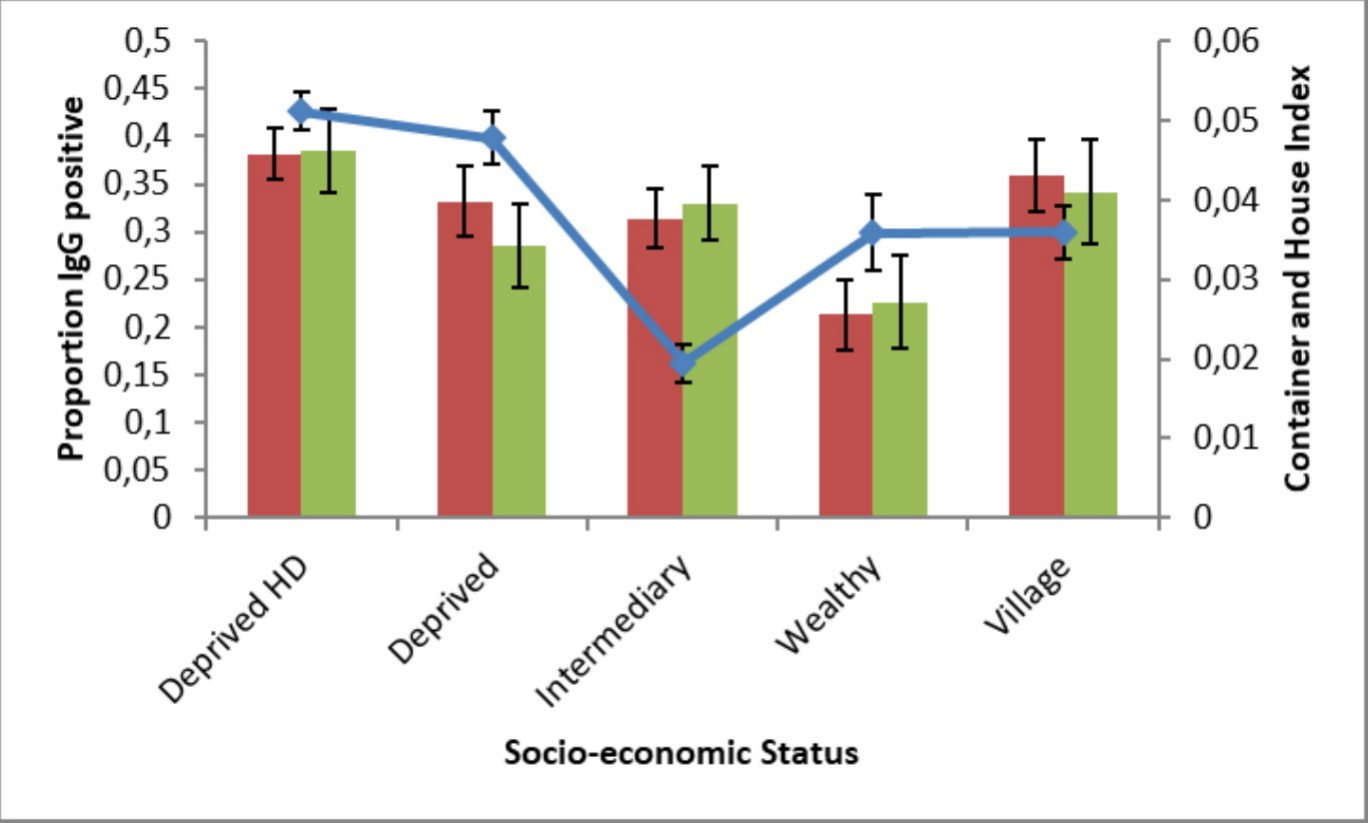
Container (green bars) and House (red bars) Indices (proportion of containers positive per house and houses positive for mosquito larvae respectively) (means and standard errors) and IgG prevalence rates (blue line, means and binomial errors) by SES typology.

**Fig 3:**
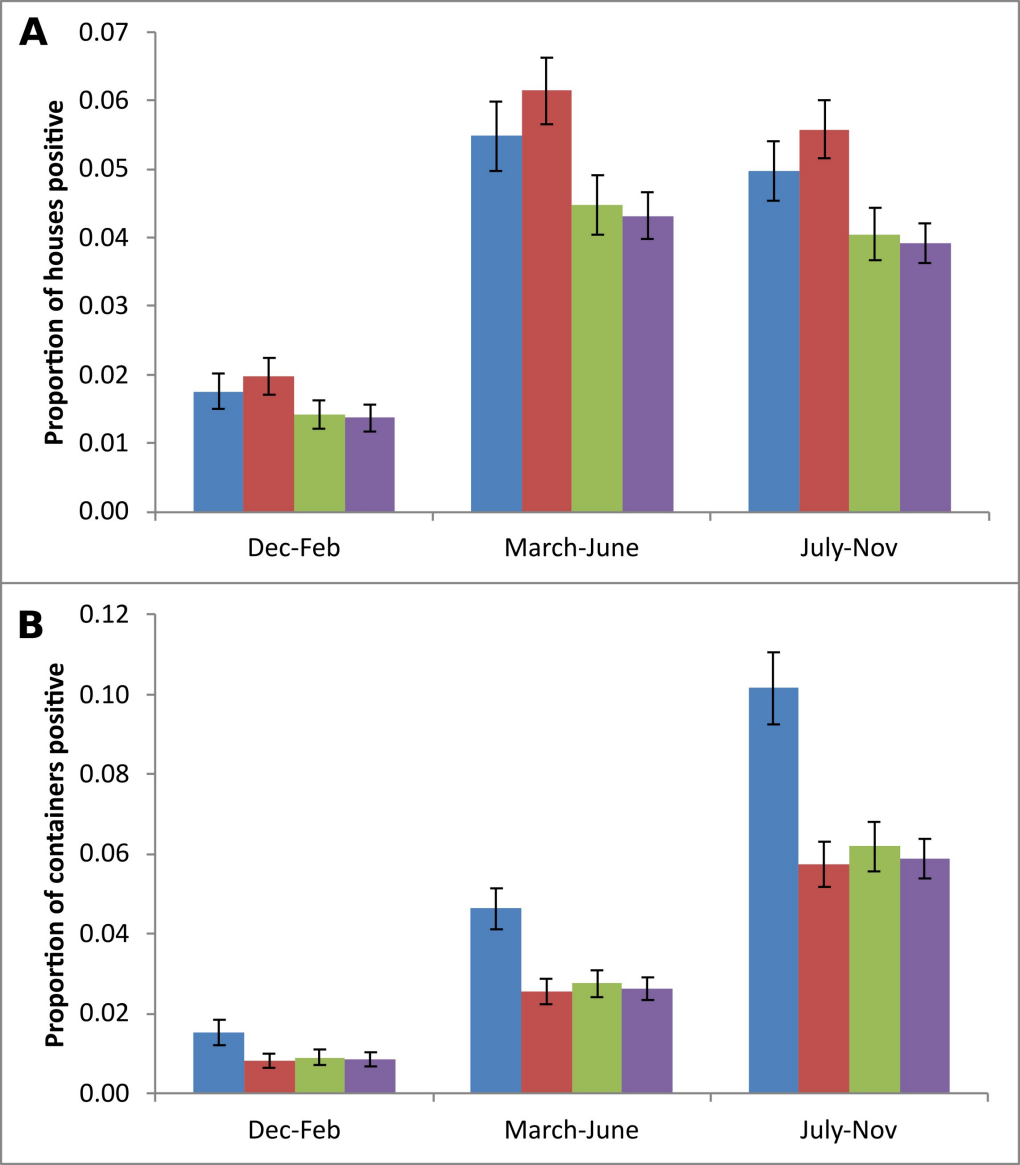
House Index and Container Index by season and Tap water access categories (Blue: 24–60%; Red: 61–92%; Green: 93–98%; Mauve: >98%). Shown are Means and standard errors.

Monthly entomological investigations in randomly selected houses within the colonies led to House indices (HI) and Container indices (CI) to be generated for 14,681 houses and 25,643 containers. Both mosquito larval indices were significantly lower in Wealthy colonies as compared to Deprived HD colonies (HI, aOR 0.55 95% C.I. 0.41–0.74, P<0.001; CI, aOR 0.54 95% C.I. 0.38–0.78, P = 0.001) (Fig 2). Deprived HD also had marginally higher HI than Intermediary colonies (aOR 0.79 95% C.I. 0.65–0.96, P = 0.017) and marginally higher CI than Deprived colonies (aOR 0.70 95% C.I. 0.53–0.92, P = 0.01). As expected, there were also significant increases in larval occurrence from March to November as compared to the winter months December to February. Colonies with less than 61% access to tap water had higher abundances of mosquito larvae, whether measured by House or Container Index, throughout the year (Fig 3A and 3B).

Focussing specifically on Deprived HD and Deprived settings, a Knowledge, Attitudes and Practices and serological studies were carried out at the family level. Only 8 individuals out of the 338 tested were found to be IgM/NS1 positive and 62 were positive for IgG. There were too few IgM positive individuals for statistical analyses. However, for IgG positive individuals, there was a lower risk of previous DENV infection associated with access to tap water every day (aOR 0.43, 95% C.I. 0.23–0.83, P = 0.01) and use of mosquito repellent (aOR 0.52, 95% C.I. 0.29–93, P = 0.03). Neither age group (P = 0.21) nor SE typology (P = 0.51) were found to be associated with IgG positivity. No other socio-economic variables were associated with anti-DENV IgG seropositivity (S5 Table).

**Fig 4:**
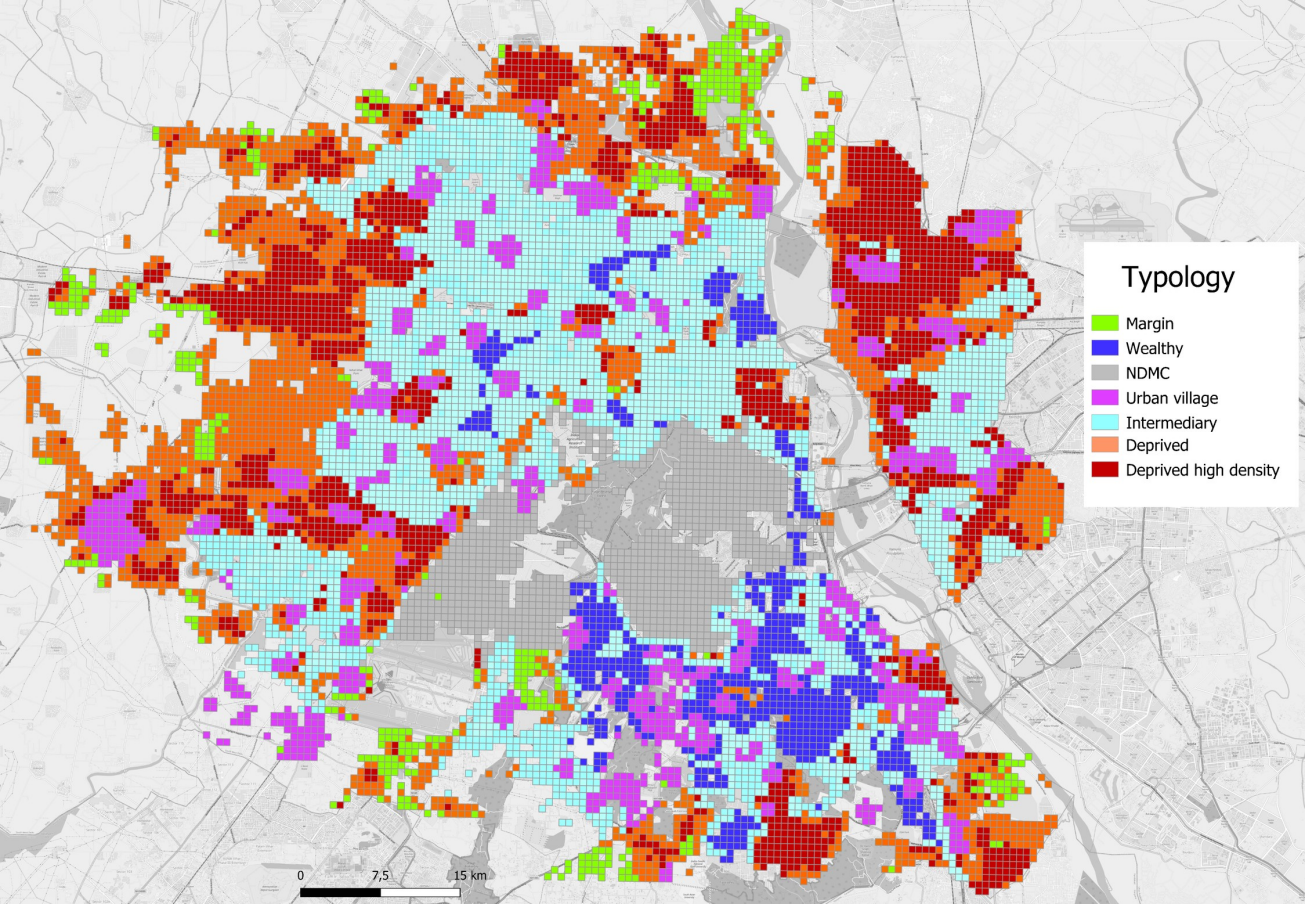
Socio-economic typology of colonies in Delhi (source Telle Olivier and OpenStreetMap). NDMC—New Delhi Municipal Corporation.

**Fig 5:**
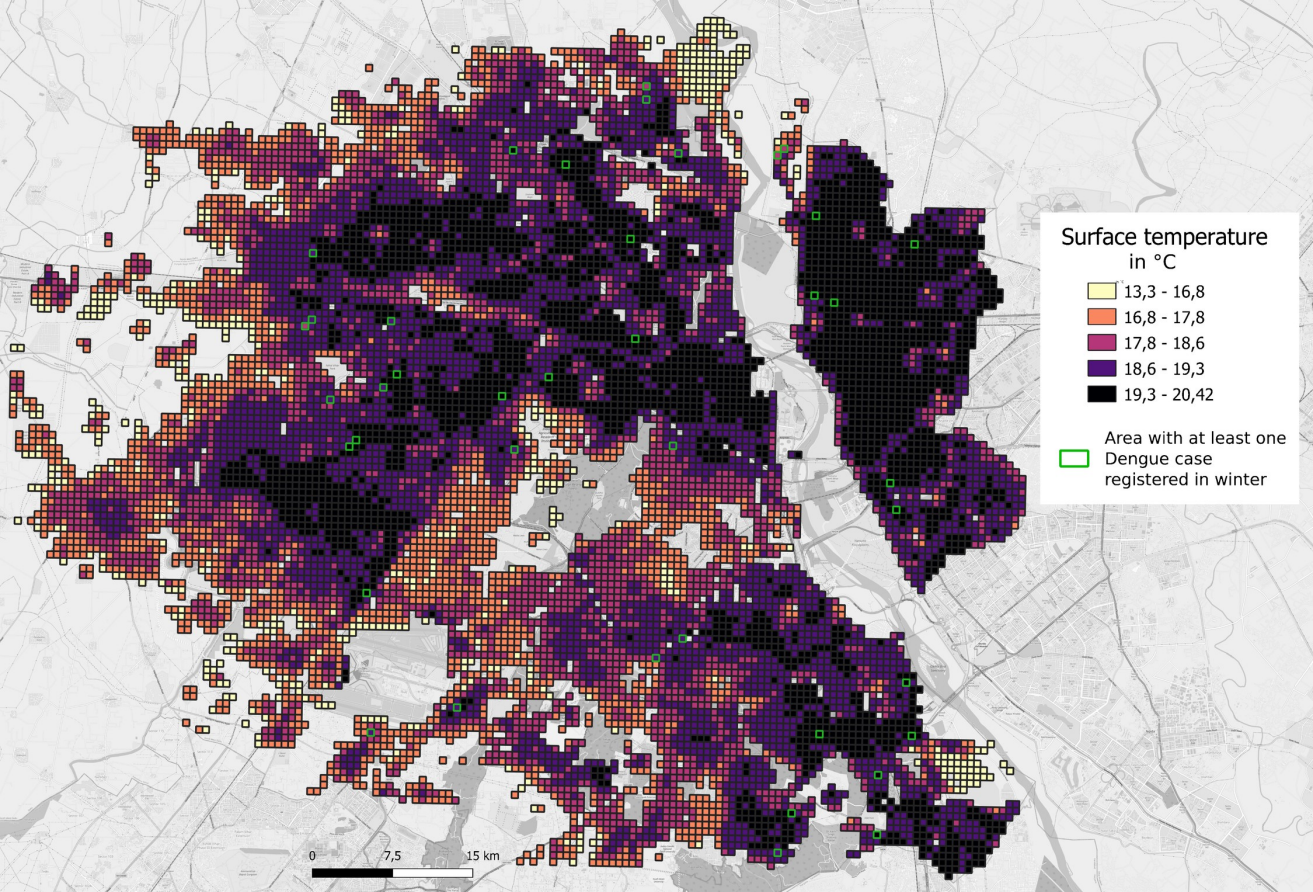
November night-time temperatures and location of the winter dengue cases.

Geolocalisation of dengue cases during the 2008, 2009 and 2010 epidemics revealed several interesting features of the epidemic trajectory and magnitude according to SE typology. Firstly, Deprived HD colonies were always the first to have cases and Wealthy colonies always later (S3–S5 Figs). Secondly, incidence rates (per 100,000 individuals) were highest in Deprived colonies, lower but comparable in Wealthy and Deprived HD colonies and lowest in Intermediary colonies (S6 and S7 Figs). We then geolocalised the first ever dengue cases observed in winter in Delhi, 2013–2015. In light of the increasing temperatures with population density, we sub-divided Deprived into Low and Medium densities and Intermediary into Intermediary High and Intermediary categories. The vast majority (32 of 41) of the cases occurred within high population density colonies of Deprived and Middle Income/Intermediary SE typology (Figs 4 and 5 and Table 3). These high-density colonies had the highest November night-time temperatures being on average in excess of 19°C and a far higher percentage of built-up areas as compared to other SE typology colonies.

**Table 3:** Risk of winter dengue cases in 250 m x 250 m units associated with Socio-economic typology.

SE typology	Dengue cases	Mean population size	Mean T°C	Built-up area (%)	RR	95% CI	P
Deprived High Density	19	2395	19.1	96.4	REF		
Deprived Low Density	0	257	17.2	19.9	1.5x10^-3^	7.6x10^-6^–0.28	0.015
Deprived Medium Density	1	948	18.0	62.9	0.24	0.16–0.36	< .001
Wealthy	1	860	18.7	65.2	0.30	0.20–0.45	< .001
Intermediary Income	0	766	18.2	48.7	8.2x10^-4^	1.6x10^-5^–0.04	< .001
Intermediary Income High Density	13	1679	19.2	93.0	0.97	0.84–1.11	0.657
NDMC	2	313	17.8	38.8	0.87	0.65–1.17	0.354
Peripheral	0	447	17.2	38.0	1.5x10^-3^	8.4x10^-6^–0.27	0.014
Urban village	5	1478	18.8	77.8	0.74	0.60–0.89	0.002

RR–Relative Risk; 95%CI—95% Confidence Intervals. Also shown are mean November night-time temperatures and % area built-up.

## Discussion

Combining sero-prevalence studies with entomological surveys in the context of socio-economic typology, we found that access to tap water was an important risk factor for dengue. Tap water access was notably lower in socially disadvantaged areas. The KAP study within the two most socially disadvantaged categories showed that this relationship holds even within these socio-economically most deprived colonies. Thus, lack of constant access to tap water rather than poverty *per se* was a risk factor for dengue infection, as found previously [28]. The lack of access to tap water likely results in more water bearing container stores in homes that could breed mosquitoes due to the storage of clean water in jars. This was supported by the random survey of houses, which showed that the most deprived areas also had poorest water access and had the highest proportion of containers positive for mosquitoes.

We also revealed a paradoxical pattern of high DENV sero-positivity in socio-economically advantaged areas despite low exposure to mosquitoes. The relationship between mosquito density and risk of dengue has long been recognised as difficult to demonstrate, not least because of the confounding effect of herd immunity and the difference between place of infection and place of illness. Furthermore, the force of infection depends intimately upon the relationship between mosquito abundance and human population density. In contrast to general assumptions that there is either a constant ratio between mosquito and human numbers (e.g. [29,30]) or their complete independence [31], mosquito numbers may increase more or less proportionally to the human population with concomitant effects on the force of infection [32,33]. At very high human densities, mosquito densities may be proportionally lower because of the lack of available breeding sites [28]. This would result in a lower force of infection at high human densities: a dilution effect. Conversely, at lower human densities, mosquito densities may initially increase more than proportionally leading to increased epidemic risk despite relatively low human densities [28,33]. The extent to which the force of infection becomes saturated at very high mosquito densities as is the case for malaria remains unknown but a possibility [34]. Recently, longitudinal entomological surveys have shown that mosquito densities do positively associate with dengue seroprevalence [35]. This makes our result even more puzzling, but one explanation may be linked to within city mobility and the specific intra-urban geography of Delhi. The spread of the virus throughout the city will be influenced by human mobility and thus predominantly occur along the major axes to the places of centrality such as markets, train stations and so forth [10,19]. In Delhi, one major difference in low dengue-affected Intermediary income as compared to the Wealthy colonies, is their relative isolation. Thus, whilst mosquito densities are as high as in Deprived areas, the lack of daily commuting from high dengue risk areas (Deprived HD and Deprived) may reduce importation of virus and result in lower sero-prevalence rates. By contrast, Wealthy colonies are hubs for daily intra-urban movement [19] and the importance of intra-urban mobility in dengue diffusion may generate the paradoxically high levels of dengue despite adequate associated infra-structure and lower mosquito density. Whilst an increasing number of studies emphasise the important role intra-city people movement plays in diffusion of DENV [18–20,36,37], to our knowledge no empirical study has explicitly coupled differing mosquito densities with intra-urban movement to explain observed spatial patterns of dengue. A high level of viral importation from high-risk sources of infection could thus enable a relatively high force of infection despite modest mosquito densities.

The significance of the Deprived HD areas as a source of infection was again revealed through the surveillance data from 2009 to 2010, which revealed a striking seasonal phenomenon. Inter-annual spatial correlation of dengue cases revealed that 35% of the first 100 cases in 2010 occurred within 200 metres of dengue clusters from the previous season in 2009 (data from [10]) (S6 Table). These cases occurred in the Deprived and high population density areas, suggesting local persistence of dengue despite global temperatures being too cold for dengue transmission. Indeed, temperatures within the city in winter can vary by over 10°C across the city and winter urban heat islands occur in such socially disadvantaged areas, offering the potential for dengue transmission at a very local level and persistence of vectors in these areas [14]. This was confirmed with the very first dengue cases registered in Delhi in winter by the surveillance system in 2013, 2014 and 2015, and the majority were reported within urban hotspots that were high density areas (Figs 4 and 5). The existence of such recognisable and potentially predictable reservoirs of infection offers a real possibility of successful, targeted mosquito control. Strategies based on improved access to tap water plus focal intervention in intra-urban hotspots prior to the dengue season could not only lead to a reduction in mosquito abundance but also eliminate the reservoir of dengue virus now circulating at low levels in winter.

There are several limitations to this study. The first concerns the SES classification of the selected colonies and the generalisability of such classifications to cities outside of India. Classifications based on wealth indicators will always be subjective to an extent, but do offer at least some quantitative indication of SE status. However, residual confounding cannot be ruled out in our study and SE typology may still be important. The majority of cities worldwide do have wealth indicators, albeit differing from the ones used here. The second limitation is the application of colony level indicators to household level sero-positivity profiles. There will always be heterogeneity in SE and environmental parameters within a colony, but use of colony level indicators is a first step and enables a broad comparison of factors associated with risk of dengue. However, as a second step, in recognition of such heterogeneity, this study also examined associations with household level factors. That at both spatial levels lack of access to tap water was identified as being associated with increased risk of dengue virus infection lends credence to its association. Another limitation of our study was the use of larval mosquito indices as a measure mosquito density. Despite their recommended use by WHO [1] for surveillance programs, they have been shown to have limited predictive value for dengue incidence [32,38]. Whilst adult mosquito densities would be expected to better predict dengue incidence, being of direct epidemiological importance, even these were not found to do so in cross-sectional studies [35]. A final limitation is the relatively small number of recruited individuals, especially in the Wealthy category, where refusal to participate was the most common. Although anticipated, we were not able to convince sufficient numbers of individuals.

The first great urbanization followed the industrial revolution in the 18th century and contagious diseases, most notably cholera, swept through the cities, affecting the most deprived population along with the wealthier population. The great sanitary awakening that preceded germ theory was a strong environmental hygiene response that led to complete remodelling of major cities such as Chicago and Paris [39]. Haussmann transformed Paris by destroying the medieval unsanitary housing and re-building along wide, spacious avenues. Chicago undertook the most ambitious sanitary engineering project of reversing the flow of the Chicago River to carry all sewage into the Illinois River. Currently, we are witness to another great urbanization, most notably in Asia and South America and a concomitant inexorable spread of diseases. However, city planning and improved infrastructure for all is no longer considered within a disease mitigation perspective. There is, however, no need to entirely re-structure our cities as in the 19th century, but simply invest in the most fundamental of human rights, access to tap water [40]. Such basic access to tap water could not only reduce the burden of dengue but also epidemics caused by other water-related diseases such as cholera [41]. The spread of disease recognizes no socio-economic boundaries and eliminating sources of infection must be a priority, not only for the socially disadvantaged population, but for the urban community as a whole. In conclusion, there is an important need to put health at the centre of urban planning and not least provide access to tap water.

## Supporting information

S1 Table Summary of number of colonies, population size and density according to Socio-economic status typology. (DOCX)

S2 Table Characteristics of colonies sampled. (DOCX)

S3 Table Socio-economic factors from the 18 study colonies. (DOCX)

S4 Table Characteristics of 338 participants from 152 participating houses in the KAP study. (DOCX)

S5 Table Univariate analyses of KAP variables for IgG sero-positivity. In bold those variables fitted in the multivariate GLMM (family as the random factor).
(DOCX)

S6 Table Distance of first 100 2010 dengue cases and all 2010 dengue cases from dengue clusters occurring in 2009. (DOCX)

S1 Fig Number of colonies per property tax score in bins of 4 units. Each bin number corresponds to the lowest value of the bin.
(DOCX)

S2 Fig Population density map of Delhi. (DOCX)

S3 Fig Dengue cases recorded by the Delhi surveillance system in 2008 classified according to Socio-economic typology. Deprived High density–Black; Deprived–Green; Intermediary–Turquoise; High category–Blue; Village–Red; NDMC–Pink; Peripheral–Yellow.
(DOCX)

S4 Fig Dengue cases recorded by the Delhi surveillance system in 2009 classified according to Socio-economic typology. Deprived High density–Black; Deprived–Green; Intermediary–Turquoise; High category–Blue; Village–Red; NDMC–Pink; Peripheral–Yellow.
(DOCX)

S5 Fig Dengue cases recorded by the Delhi surveillance system in 2010 classified according to Socio-economic typology. Deprived High density–Black; Deprived–Green; Intermediary–Turquoise; High category–Blue; Village–Red; NDMC–Pink; Peripheral–Yellow.
(DOCX)

S6 Fig Dengue case incidence rates per 100,000 individuals recorded by the Delhi surveillance system in 2008 classified according to Socio-economic typology. Deprived High density–Black; Deprived–Green; Intermediary–Turquoise; High category–Blue; Village–Red; NDMC–Pink; Peripheral–Yellow.
(DOCX)

S7 Fig Dengue case incidence rates per 100,000 individuals recorded by the Delhi surveillance system in 2010 classified according to Socio-economic typology. Deprived High density–Black; Deprived–Green; Intermediary–Turquoise; High category–Blue; Village–Red; NDMC–Pink; Peripheral–Yellow.
(DOCX)
