# Supplementary material for: Social and environmental risk factors for dengue in Delhi city: A retrospective study
Source: PLoS Negl Trop Dis. 2021 Feb 11;15(2):e0009024. doi: 10.1371/journal.pntd.0009024 (PMC7877620; doi:10.1371/journal.pntd.0009024)
Supplement: S1 Table — (DOCX) [file pntd.0009024.s001.docx]

**S1 Table. Summary of number of colonies, population size and density according to Socio-economic status typology.**

| **Typology** | **Property Tax score** | **Share (units)** | **Total population** | **Share population** | **Mean Density km²** |
| --- | --- | --- | --- | --- | --- |
| Deprived HD | 0<40 | 28.50% | 6 410 310 | 43.30% | 42 816 |
| Deprived | 0<40 | 15.70% | 975 596 | 6.60% | 8 752 |
| Intermediary | 40<65 | 34.56% | 4 446 582 | 30.50% | 19 856 |
| Higher category | >65 | 8.60% | 652 123 | 4.40% | 14 080 |
| NDMC | Not included | 6.60% | 289 149 | 2.00% | 34 816 |
| Urban village | Special category | 4.10% | 1 808 850 | 12.20% | 23 648 |
| Peripherical | 0 | 2.00% | 212 000 | 1.00% | 264 |
