# Supplementary material for: Social and environmental risk factors for dengue in Delhi city: A retrospective study
Source: PLoS Negl Trop Dis. 2021 Feb 11;15(2):e0009024. doi: 10.1371/journal.pntd.0009024 (PMC7877620; doi:10.1371/journal.pntd.0009024)
Supplement: S2 Table — (DOCX) [file pntd.0009024.s002.DOCX]

**S2 Table. Characteristic of colonies sampled.**

| **ID** | **Property tax**  **score** | **Type** | **House in good shape** | **Share of HH with more than 5 Individuals** | **Source of drinking = Tapwater from treated source (%)** | **Main water acces within premises (%)** | **HH with**  **no drainage**  **accessibility**  **(%)** | **Pop /Km²** |
| --- | --- | --- | --- | --- | --- | --- | --- | --- |
| 1 | 30 | Deprived | 71.70 | 48.70 | 59.40 | 68.30 | 0.20 | 18 480 |
| 2 | 32 | Deprived | 68.80 | 40.00 | 92.90 | 74.50 | 3.10 | 23 136 |
| 3 | 20 | Deprived | 56.70 | 52.30 | 98.50 | 93.50 | 0.30 | 26 656 |
| 4 | 20 | Deprived | 39.90 | 58.20 | 15.10 | 14.60 | 28.00 | 17 936 |
| 5 | 50 | Deprived HD | 65.10 | 46.80 | 95.90 | 98.40 | 0.00 | 73 280 |
| 6 | 32 | Deprived HD | 78.00 | 45.40 | 91.90 | 85.60 | 2.10 | 64 928 |
| 7 | 20 | Deprived HD | 63.40 | 52.90 | 49.60 | 56.60 | 4.10 | 52 000 |
| 8 | 20 | Deprived HD | 69.40 | 44.80 | 53.90 | 82.80 | 6.30 | 58 896 |
| 9 | 28 | Deprived HD | 52.50 | 57.90 | 17.30 | 45.10 | 34.20 | 58 976 |
| 10 | 76 | Wealthy | 91.90 | 39.00 | 99.70 | 99.80 | 0.50 | 18 016 |
| 11 | 72 | Wealthy | 85.40 | 38.70 | 85.70 | 91.20 | 1.70 | 18 624 |
| 12 | 64 | Intermediary | 48.90 | 45.70 | 88.20 | 70.30 | 1.60 | 41 968 |
| 13 | 50 | Intermediary | 76.40 | 42.80 | 92.50 | 93.40 | 0.50 | 23 136 |
| 14 | 48 | Intermediary | 44.10 | 50.50 | 90.90 | 77.10 | 7.50 | 32 288 |
| 15 | 52 | Intermediary | 77.40 | 39.50 | 94.80 | 81.70 | 0.00 | 25 792 |
| 16 | 0 | Village | 80.50 | 30.40 | 87.00 | 85.30 | 3.80 | 79 168 |
| 17 | 0 | Village | 54.80 | 56.90 | 29.80 | 67.20 | 30.00 | 35 744 |
| 18 | 0 | Village | 58.20 | 54.20 | 39.50 | 53.70 | 13.50 | 34 272 |
