# Supplementary material for: Social and environmental risk factors for dengue in Delhi city: A retrospective study
Source: PLoS Negl Trop Dis. 2021 Feb 11;15(2):e0009024. doi: 10.1371/journal.pntd.0009024 (PMC7877620; doi:10.1371/journal.pntd.0009024)
Supplement: S3 Table — (DOCX) [file pntd.0009024.s003.docx]

S3 Table. Socio-economic factors from the 18 study colonies

| **Information at Colony scale** | N | % |
| --- | --- | --- |
| **Type of house** |  |  |
| Good | 1430 | 67.9 |
| liveable | 590 | 28.0 |
| dilapidated | 87 | 4.1 |
| **Household size** |  |  |
| 1 | 83 | 3.9 |
| 2 | 167 | 7.9 |
| 3 | 296 | 14.0 |
| 4 | 549 | 26.1 |
| 5 | 422 | 20.0 |
| 6 to 8 | 505 | 24.0 |
| 9 | 85 | 4.0 |
| **Tap water Access (%)** |  |  |
| 24-59.7 | 695 | 33.0 |
| 59.8-91.3 | 424 | 20.1 |
| 91.4-98.4 | 548 | 26.0 |
| 98.5-99.9 | 444 | 21.1 |
| **Main source of drinking** |  |  |
| Tapwater from treated source | 1496 | 71.0 |
| Tapwater from un-treated source | 174 | 8.3 |
| Covered well | 8 | 0.4 |
| Un-covered well | 0 | 0.0 |
| Handpump | 55 | 2.6 |
| Tubewell/Borehole | 231 | 11.0 |
| Spring | 0 | 0.0 |
| River/ | 0 | 0.0 |
| Canal | 0 | 0.0 |
| Tank/ | 29 | 1.4 |
| Pond/ | 0 | 0.0 |
| Lake | 0 | 0.0 |
| Other sources | 114 | 5.4 |
| **Place of main source of drinking water** |  |  |
| Within premises | 1559 | 74.0 |
| Near premises | 379 | 18.0 |
| Further away | 169 | 8.0 |
| **Number of households having latrine facility** |  |  |
| Yes | 1889 | 89.7 |
| No | 218 | 10.3 |
| **Waste water outlet connected to** |  |  |
| Closed drainage | 1430 | 67.9 |
| Open drainage | 548 | 26.0 |
| No drainage | 129 | 6.1 |
