# Supplementary material for: Social and environmental risk factors for dengue in Delhi city: A retrospective study
Source: PLoS Negl Trop Dis. 2021 Feb 11;15(2):e0009024. doi: 10.1371/journal.pntd.0009024 (PMC7877620; doi:10.1371/journal.pntd.0009024)
Supplement: S4 Table — (DOCX) [file pntd.0009024.s004.docx]

S4 Table. Characteristics of 338 participants from 152 participating houses in the KAP study. INR – Indian Rupee.

| Age (Median) | **26** |  |
| --- | --- | --- |
|  | **N** | % |
| **Sex** |  |  |
| Female | 207 | 61.2 |
| Male | 131 | 38.8 |
| **Typology colony** |  |  |
| Deprived Low | 227 | 67.2 |
| Deprived | 111 | 32.8 |
| **KAP Individual** |  |  |
| Outside Delhi in the last 10 days | 19 | 5.6 |
| Vaccinate for Yellow Fever | 0 | 0.0 |
| **KAP Family** |  |  |
| **Income category** |  |  |
| 1 (4,000-7,500 INR) | 57 | 37.5 |
| 2 (8,000-13,000 INR) | 37 | 24.3 |
| 3 (15,000-27,000 INR) | 52 | 34.2 |
| No information | 6 | 3.9 |
| **Live in Delhi since** |  |  |
| 1 (0-9 yrs) | 40 | 26.3 |
| 2 (10-16 yrs) | 41 | 27.0 |
| 3 (+16 yrs) | 70 | 46.1 |
| No information | 1 | 0.7 |
| **Construction (brick)** |  |  |
| Brick | 150 | 98.7 |
| Temporary | 2 | 1.3 |
| **Floor of building** |  |  |
| Ground | 92 | 60.5 |
| First | 21 | 13.8 |
| Second | 24 | 15.8 |
| Third | 5 | 3.3 |
| Above third | 11 | 7.2 |
| *Note one family lived on two floors | |  |
| **Type of House** |  |  |
| Building | 51 | 33.6 |
| Individual | 101 | 66.4 |
| **Behavior** |  |  |
| Are pestered by mosquitoes | 118 | 77.6 |
| Use Repellent at least twice a week | 105 | 69.1 |
| Have window screens | 59 | 38.8 |
| Have access to tap water | 67 | 44.1 |
| Use Air Cooler | 114 | 75.0 |
| Use AC | 8 | 5.3 |
| Occupation at Home | 15 | 9.9 |
| **Family exposure to dengue virus** |  |  |
| At least one IgG positive in the family | 52 | 34.2 |
