# Supplementary material for: Social and environmental risk factors for dengue in Delhi city: A retrospective study
Source: PLoS Negl Trop Dis. 2021 Feb 11;15(2):e0009024. doi: 10.1371/journal.pntd.0009024 (PMC7877620; doi:10.1371/journal.pntd.0009024)
Supplement: S5 Table — In bold those variables fitted in the multivariate GLMM (family as the random factor). (DOCX) [file pntd.0009024.s005.docx]

**S5 Table.** Univariate analyses of KAP variables (in S2 Table) for IgG sero-positivity. In bold those variables fitted in the multivariate GLMM (family as the random factor).

|  | P value |
| --- | --- |
|  |  |
|  |  |
| **Age group (3 categories)** | **0.124** |
| Gender (F/M) | 0.759 |
| Construction (brick or temporary) | 0.772 |
| Floor (of building 5 storeys) | 0.366 |
| Type of House (building or individual) | 0.413 |
| Movement outside Delhi in last 10 days (Y/N) | 0.359 |
| **Live in Delhi (0-9yrs, 10-16 yrs, 16+ years)** | 0.578 |
| **Pestered by mosquitoes (Y/N)** | 0.509 |
| **Use of Repellent (Y/N)** | **0.003** |
| **Type of Water supply (Tap water Y/N)** | **0.005** |
| A/C (Y/N) | 0.851 |
| Cooler (Y/N) | 0.484 |
| Windows/doors with nets (Y/N) | 0.594 |
| Work from home (Y/N) | 0.999 |
| **Income category (3 categories)** | **0.229** |
| **Other dengue case in family (Y/N)** | **0.211** |
