# Supplementary material for: Social and environmental risk factors for dengue in Delhi city: A retrospective study
Source: PLoS Negl Trop Dis. 2021 Feb 11;15(2):e0009024. doi: 10.1371/journal.pntd.0009024 (PMC7877620; doi:10.1371/journal.pntd.0009024)
Supplement: S6 Table — (DOCX) [file pntd.0009024.s006.docx]

**S6 Table.** Distance of first 100 2010 dengue cases and all 2010 dengue cases from dengue clusters occurring in 2009

| distance (m) from dengue clusters in 2009 | First 100 cases 2010 | All cases 2010 |
| --- | --- | --- |
| <200 | 35 | 1575 |
| 200/500 | 22 | 1196 |
| 500/1000 | 13 | 1277 |
| 1000/1500 | 15 | 731 |
| 1500+ | 15 | 1209 |
