# Supplementary material for: Social and environmental risk factors for dengue in Delhi city: A retrospective study
Source: PLoS Negl Trop Dis. 2021 Feb 11;15(2):e0009024. doi: 10.1371/journal.pntd.0009024 (PMC7877620; doi:10.1371/journal.pntd.0009024)

**S1 Fig** Number of colonies per property tax score in bins of 4 units. Each bin number corresponds to the lowest value of the bin.


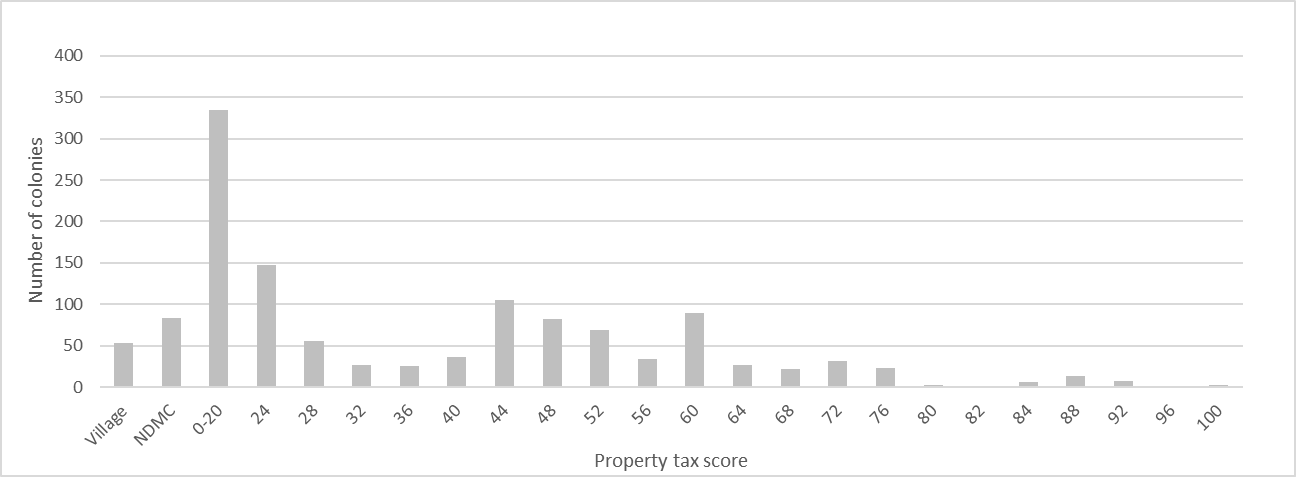

Supplement: S1 Fig — Each bin number corresponds to the lowest value of the bin. (DOCX) [file pntd.0009024.s007.docx]
