# Supplementary figures and images for: Social and environmental risk factors for dengue in Delhi city: A retrospective study
Source: PLoS Negl Trop Dis. 2021 Feb 11;15(2):e0009024. doi: 10.1371/journal.pntd.0009024 (PMC7877620; doi:10.1371/journal.pntd.0009024)

**S2 Fig** Population density map of Delhi


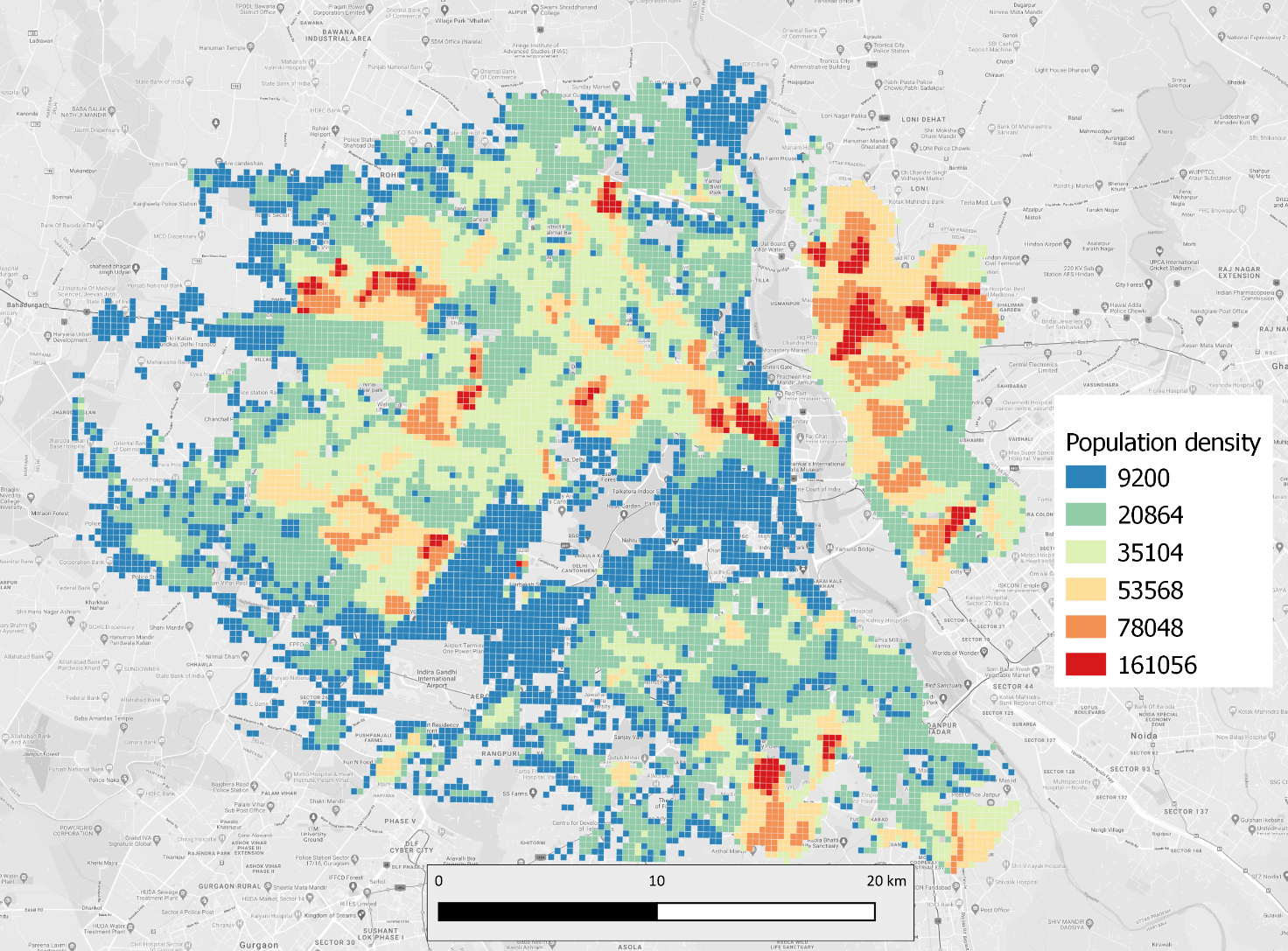

Supplement: S2 Fig — (DOCX) [file pntd.0009024.s008.docx]
