# Supplementary material for: Social and environmental risk factors for dengue in Delhi city: A retrospective study
Source: PLoS Negl Trop Dis. 2021 Feb 11;15(2):e0009024. doi: 10.1371/journal.pntd.0009024 (PMC7877620; doi:10.1371/journal.pntd.0009024)

**S6 Fig** Dengue case incidence rates per 100,000 individuals recorded by the Delhi surveillance system in 2008 classified according to Socio-economic status. Deprived High density – Black; Deprived – Green; Intermediary – Turquoise; High category – Blue; Village – Red; NDMC – Pink; Peripheral – Yellow.


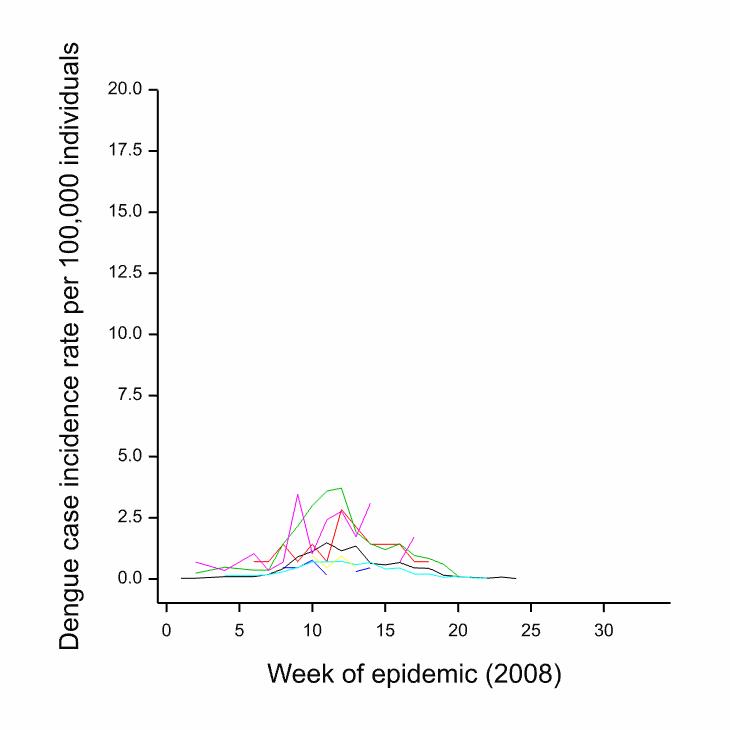

Supplement: S6 Fig — Deprived High density–Black; Deprived–Green; Intermediary–Turquoise; High category–Blue; Village–Red; NDMC–Pink; Peripheral–Yellow. (DOCX) [file pntd.0009024.s012.docx]
